# Juxtaposition of System Dynamics and Agent-Based Simulation for a Case Study in Immunosenescence

**DOI:** 10.1371/journal.pone.0118359

**Published:** 2015-03-25

**Authors:** Grazziela P. Figueredo, Peer-Olaf Siebers, Uwe Aickelin, Amanda Whitbrook, Jonathan M. Garibaldi

**Affiliations:** 1 School of Computer Science, University of Nottingham, Jubilee Campus, Nottingham, United Kingdom; 2 Advanced Data Analysis Centre, University of Nottingham, United Kingdom; 3 Department of Computer Science, Loughborough University, Epinal Way, Loughborough, United Kingdom

## Abstract

Advances in healthcare and in the quality of life significantly increase human life expectancy. With the aging of populations, new un-faced challenges are brought to science. The human body is naturally selected to be well-functioning until the age of reproduction to keep the species alive. However, as the lifespan extends, unseen problems due to the body deterioration emerge. There are several age-related diseases with no appropriate treatment; therefore, the complex aging phenomena needs further understanding. It is known that immunosenescence is highly correlated to the negative effects of aging. In this work we advocate the use of simulation as a tool to assist the understanding of immune aging phenomena. In particular, we are comparing system dynamics modelling and simulation (SDMS) and agent-based modelling and simulation (ABMS) for the case of age-related depletion of naive T cells in the organism. We address the following research questions: Which simulation approach is more suitable for this problem? Can these approaches be employed interchangeably? Is there any benefit of using one approach compared to the other? Results show that both simulation outcomes closely fit the observed data and existing mathematical model; and the likely contribution of each of the naive T cell repertoire maintenance method can therefore be estimated. The differences observed in the outcomes of both approaches are due to the probabilistic character of ABMS contrasted to SDMS. However, they do not interfere in the overall expected dynamics of the populations. In this case, therefore, they can be employed interchangeably, with SDMS being simpler to implement and taking less computational resources.

## Introduction

Bulati *et al*. [1] characterises ageing as a complex process with negative repercussion on the development and functioning of the immune system. Progressive changes of the immune system components have a major impact on the capacity of an individual to produce effective immune responses. The decrease of immunocompetence in the elderly is the result of the continuous challenge and unavoidable exposure to a variety of potential antigens [2]. Virus, bacteria, fungus, food, etc. cause persistent life-long antigenic stress, and immunological space is filled with effector cells and immune memory cells.

Franceschi [2] also identified some factors that characterise immune system ageing, many of which affect immune cell populations such as T cells. There is, for instance an accumulation of memory T cells and a marked reduction of the T cell repertoire. In addition, thymic involution, which occurs after the twenties, has a significant impact in the reduction of naïve T cells numbers. Naïve T cells are immunocompetent cells that respond to previously unencountered antigens. The depletion of these cells therefore eventually leaves the body more susceptible to new diseases [3].

This work is an extension of initial experiments with naïve T cell populations presented in [4]. Our objective is to study the decay of these cell numbers with age, under a simulation perspective. We therefore compare SDMS and ABMS for an immune system ageing model involving interactions that influence the naïve T cell populations over time. The model is based on a set of ordinary differential equations (ODEs) defined in [3]. In their work, Murray *et al*. [3] propose a model with a set of equations to fit observed data and estimate the output of different naïve T cells populations with age. We build equivalent models employing SDMS and ABMS, compare results and validate our outcomes with real-world data and the original ODE model [3]. Our objective is to answer the following research questions: Which approach is more suitable for this problem? Can we use both techniques interchangeably? What are the benefits and problems encountered in each method for this problem? In order to answer our research questions the naïve depletion case study is investigated under five scenarios defined in [3]. For each setting, the rates of changes within the cell populations are modified in order to determine how these changes are reflected under each simulation technique.

### Background

A computational simulation of a system can be defined as an “*imitation (on a computer) of a system as it progresses through time*” [5]. Its purpose is to understand, change, manage and control reality [6]. Moreover, simulation is employed to obtain further understanding of a system and/or to identify improvements to a system [5]. A simulation predicts the performance of a system given a specific set of inputs. According to Robinson [5], simulation is an experimental approach to modelling a “*what-if*” analysis tool. The model user determines the scenarios and the simulation predicts the outcomes. Simulation can, therefore, also be seen as a decision support tool. Compared to real-world experimentation, simulation is generally more cost-effective and less time consuming. Furthermore, under a controlled simulation environment, changes and different scenarios are analysed without impact to the real-world [5], requiring no ethical agreements. The choice of the appropriate simulation method [4, 7, 8] will determine the efficacy of the decision tool for a certain problem. In addition, different approaches impact on the content of the information produced by the simulation. Current major system simulation modelling methods include system dynamics modelling (SDM), discrete-event modelling, dynamic systems modelling and agent-based modelling (ABM) [9]. We however investigate SDM and ABM, as they appear to be the most employed in immunology [10].

System Dynamics (SD) [12] is an aspect of systems theory currently applied to any complex system characterized by interdependency, mutual interaction, information feedback and circular causality. The basis of the SD methodology is the recognition that the structure of a system is just as important in determining its behaviour as the individual components themselves. It is therefore necessary to adopt a “systemic way of thinking” [13], where the focus is on the top-down, internal system structure. This means that the problem should be depicted as a set of patterns, interrelated processes and generic structures [14]. SD works with feedback loops, stocks and flows that describe a system’s nonlinearity. The changes that occur over time in the variables of the problem are ruled by ODEs. After understanding the structure of the problem to be simulated, it is necessary to translate it into a causal loop diagram, which is a graphical representation used in SD. Causal loop diagrams aid visualization of how interrelated variables affect one another. System Dynamics Simulation (SDS) is a continuous simulation for an SD model. It consists of a set of ODEs that are solved for a certain time interval [15]. The simulation, therefore, has a deterministic output.

Agent-based modelling and simulation (ABMS) is a stochastic method that employs a set of autonomous agents that interact with each other in a certain environment [16]. As it is derived from complex systems, its baseline is the notion that systems are built in a bottom-up perspective. In other words, an understanding of the dynamics of the system arises from individual interactions and their environment [17]. The agents’ behaviours are described by rules that determine how they learn, interact with each other and adapt. The overall system behaviour is given by the agents’ individual behaviours as well as by their interactions. ABMS is therefore well suited to modelling and simulating systems with heterogenous, autonomous and pro-active actors, such as human-centred systems, biological systems, businesses and organizations [18]. State chart diagrams from the unified modelling language (UML) can be used to aid agent based modelling. With state charts it is possible to define and visualize the agents’ states, transitions between the states, events that trigger transitions, timing and agent actions [9].

The next section presents a literature review of work concerned with the comparison of SDMS and ABMS for different problem domains. We initially review general work that has been carried out to assess the differences of both approaches. Subsequently, we focus on research concerned with the comparison of strategies for immunological problems. We find, however, that there is a scarcity of literature comparing the two approaches for immune simulation.

### Related Work

Scholl [19] conducts one of the first studies characterising the domains of applicability of SDMS and ABMS. The author also investigates the strengths and weaknesses of each approach, outlining opportunities for SDMS and ABMS-based multi-method modelling. Pourdehnad *et al*. [20] extends this pioneer work by comparing the two approaches conceptually. The authors discuss the potential synergy between both paradigms to solve problems of teaching decision-making processes. Similarly, Stemate *et al*. [21] compare these modelling approaches and identify a list of likely opportunities for cross-fertilization. The authors see this list as a starting point for other researchers to take such synergistic views further.

Schieritz [22] and Scheritz *et al*. [23] contribute largely to the comparison of SDMS and ABMS for operational research (OR). They identify the unique features of each approach and present a table with their main differences. Further in [24] the authors describe an approach to combine ODEs and ABMS for solving supply chain management problems. Their results show that the combined SDMS/ABMS does not produce the same outcomes as those from the SDMS alone. To understand why these differences occur, the authors indicate that more research needs to be conducted.

Ramandad *et al*. [25] compare the dynamics of ABMS and SDMS for contagious disease spread. The authors convert the ABM into an SDM and examine the impact of individual heterogeneity and different network topologies. They conclude that the deterministic SDMS yields a single trajectory for each parameter set, while the stochastic ABMS yields a distribution of outcomes. Moreover, the outcomes differ in several metrics relevant to public health.

Schieritz [26] analyses two arguments given in literature to explain the superiority of ABMS compared with SDMS for social simulation: (1) the inability of SD to represent emergence and (2) SD’s lack of individual diversity. The author points out that an agent-based approach models individuals and interactions on a lower level, implicitly taking up an individualist position of emergence; conversely, SD models social phenomena at an aggregate level. As a second part of the study, the author compares SDM and ABM for modelling species competing for resources to analyse the effects of evolution on population dynamics. The conclusion is that when individual diversity is considered, it limits the applicability of the ODE model. However, it is shown that “*a highly aggregate more ODE-like model of an evolutionary process displays similar results to the ABMS*”.

Similarly, Lorenz [8] proposes three aspects to consider when choosing between SDMS and ABMS: structure, behaviour and emergence. Structure is related to how the model is built. The structure of a SDMS model is static, whereas in ABMS it is dynamic. In SDMS, all the elements of the simulation are developed in advance. In ABMS, on the other hand, agents are created or destroyed and interactions are defined through the course of the simulation run. The second aspect (behaviour) focuses on the central generators of behaviours in the model. For SDMS the behaviour generators are feedback and accumulations, while for ABMS they are the interactions of the systems elements. Both methodologies incorporate feedback. ABMS, however, has feedback in more than one level of modelling. The third aspect relates to capacity to capture emergence, which differs between the two methodologies. In disagreement with Schieritz [26] mentioned earlier, the author states that ABMS is capable of capturing emergence, while the one-level structure of SDMS is insufficient in that respect.

In previous work [10, 11] we discuss the merits of SDMS and ABMS for problems involving the interactions with the immune system and early-stage cancer. Our interest was to identify those cases in immunology where ABMS and SDMS could be applied interchangeably without compromising the results’ usefulness. In addition, we wanted to investigate those circumstances where one approach was not able to reflect the compared approach outcomes. We found several outcome differences: (i) not everything produced by the SDMS can be observed in ABMS outcomes (e.g. no half agents, only atomic elements), which can impact on outcome similarity when the population size is small (less than 50 agents); (ii) for our case studies, ABMS contributed to additional insights; its stochastic nature and emergent behaviour meant that it could produce different results and extra patterns of behaviour. In this work we continue our investigations by applying the comparison to an immunosenescence problem, as shown next.

## Methods

### Case Study: Naïve T Cells Output

Before the age of 20, the set of naïve T cells is sustained primarily by the thymus [3]. Thymic contributions in an individual are quantified by the presence of a biological marker known as ‘T cell receptors excision circle’ (TREC), which is a circular DNA originated during the formation of the T-cell receptor. The percentage of T cells with TRECs decays with shrinkage of thymic output, activation and reproduction of naïve T cells [3]. This means that naïve T cells from the thymus have a greater percentage of TREC than those originating from other sources. In middle age, however, the thymus involutes and there is a change in the source of naïve T cells. There is a considerable reduction in thymic naïve T cell output, which means that new naïve T cells are mainly produced by existing cell reproduction (peripheral expansion). It is believed that, after twenties, the naïve T cell repertoire is also maintained by the population of existing memory cells, which have their phenotype reverted back to the naïve cells type [3].

These two new methods of naïve T cell repertoire maintenance, however, are insufficient to keep an effective defense system in the organism [3], as they do not produce new phenotypic changes in the T cells. Rather, evidence shows that they continue to fill the naïve T cell space with copies of existing cells [27], which are incapable of eliminating new antigens. The loss (death) of clones of some antigen-specific T cells therefore becomes irreversible. These age-related phenomena lead to a decay of immune performance.

Our study on the dynamics of naïve T cells over time is based on equations obtained in [3] and real-world data from [3, 28, 29]. The model objective is to investigate the likely contribution of each of the naïve T cell’s sources by comparing estimates of the presence of TREC in the cells. In the model, four populations are considered, naïve T cells from the thymus, naïve T cells from peripheral proliferation, active cells and memory cells. The mathematical model of the dynamics of the cell populations and its sustaining sources is presented next.

#### The Mathematical Model

The model proposed by Murray *et al*. [3] is described by equations 1 to 6, in which *N* is the number of naïve cells from the thymus, *N*_*p*_ is the number of naïve cells that have undergone proliferation, *A* is the number of active cells, *M* is the number of memory cells and *t* is time (in years). At the beginning of life most naïve T cells belong to the population *N*. With time naïve T cells from the thymus proliferate, which contributes to the increase of the *N*_*p*_ population. When the body faces a new threat, naïve T cells are recruited and become active (*A*). A fraction of active cells turns into memory cells (*M*).

The first differential equation describing the naïve T cell population from the thymus is:

$$\begin{array}{c} \frac{dN}{dt}=s_{0}e^{-\lambda_{t}t}\;s\left(N_{p}\right)-\left[\lambda_{n}+\mu_{n}g\left(N_{p}\right)\right]N \end{array}$$ (1)

where *s*_0_ is the thymic output value, *λ*_*t*_ is the thymic decay rate, *t* represents time in years, *s*_0_ *e* ^−*λ*_*t*_*t*^ *s*(*N*_*p*_) is the number of cells that arise from the thymus and *s*(*N*_*p*_) is the rate of export of the thymus defined by:

$$\begin{array}{c} s\left(N_{p}\right)=\frac{1}{1+\frac{{\bar{s}}^{N_{p}}}{\bar{N_{p}}}} \end{array}$$ (2)

$\bar{N_{p}}$ and $\bar{s}$ are equilibrium and scaling values respectively, established in [3]. *λ*_*n*_ *N* represents the naïve cells that become part of the naïve proliferating population, *λ*_*n*_ is the naïve proliferation rate, *μ*_*n*_ is the thymic naïve cell death rate, *μ*_*n*_ *g*(*N*_*p*_)*N* represents the naïve cell death rate and *g*(*N*_*p*_) is the death rate of cells between naïve TREC-positive and naïve TREC-negative, defined as:

$$\begin{array}{c} g\left(N_{p}\right)=1+\frac{\frac{bN_{p}}{\bar{N_{p}}}}{1+\frac{N_{p}}{\bar{N_{p}}}} \end{array}$$ (3)

The second differential equation describing the naïve T cells from proliferation is:

$$\begin{array}{c} \frac{dN_{p}}{dt}=\lambda_{n}N+\left[ch\left(N,N_{p}\right)-\mu_{n}\right]N_{p}+\lambda_{mn}M \end{array}$$ (4)

where *c* is the proliferation rate, *ch*(*N*, *N*_*p*_)*N*_*p*_ represents the naïve proliferation and *h*(*N*, *N*_*p*_) is the dilution of thymic-naïve through proliferation defined by:

$$\begin{array}{c} h\left(N,N_{p}\right)=\frac{1}{1+\frac{N+N_{p}}{\bar{N_{p}}}} \end{array}$$ (5)

*μ*_*n*_ *N*_*p*_ is the death rate of proliferation-originated naïve cells and *λ*_*mn*_ is the reversion rate from memory into *N*_*p*_.

The final differential equation for the memory cell population dynamics is:

$$\begin{array}{c} \frac{dM}{dt}=\lambda_{a}A-\mu_{m}M-\lambda_{mn}M \end{array}$$ (6)

where *λ*_*a*_ is the reversion rate into memory and *μ*_*m*_ is the death rate of memory cells.

**Fig 1:**
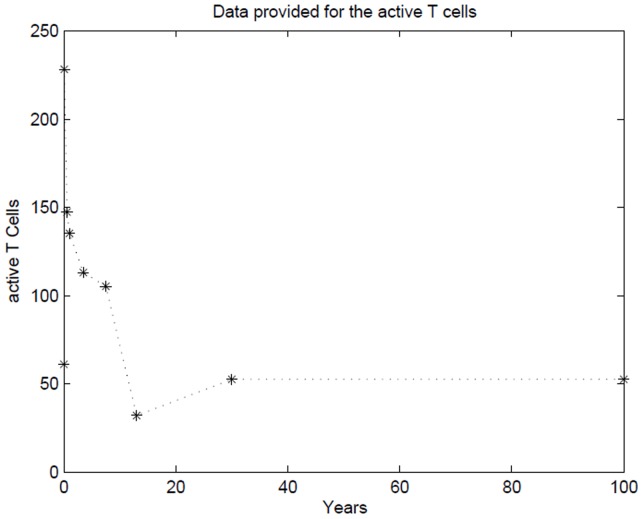
The data set used as a look-up table for the active cells.

The parameter values for the model are shown in Table 1. For the mathematical model and subsequent simulations, *s*0 = 56615. The values for active cells over time are determined by referring to data obtained in Commas-Bitter *et al*. [30] (Fig. 1). This table contains the number of activated CD4+ cells (a type of naïve T cells) per *mm* ^3^ for early years and is used as a stock for the active cells. From the active cell stock the values of the memory cell stock are updated according to the parameter *λ*_*a*_.

**Table 1:** Rate values for the mathematical model (obtained from [3]).

rate	value(s)
*λ*_*t*_	$\frac{log(2)}{15.7}(year^{-1})$
*λ*_*n*_	0.22, 2.1, 0.003
*μ*_*n*_	4.4
*c*	0 (no proliferation) or $\mu_{n}(1+\frac{300}{\bar{N_{p}}})$
*λ*_*mn*_	0
*μ*_*m*_	0.05
*λ*_*a*_	1

Equations 1 to 6 are incorporated into the SDM and ABM in order to investigate if it is possible to reproduce and validate the results obtained in [3]. Moreover, variations of the rate variables are explored to understand the importance of each individual integrand in the system. For example, it is important to establish how much the proliferation rate impacts on the depletion of naïve T cells over age, and to identify the point in time at which the system can be defined as losing functionality.

### The System Dynamics Modelling and Simulation

**Fig 2:**
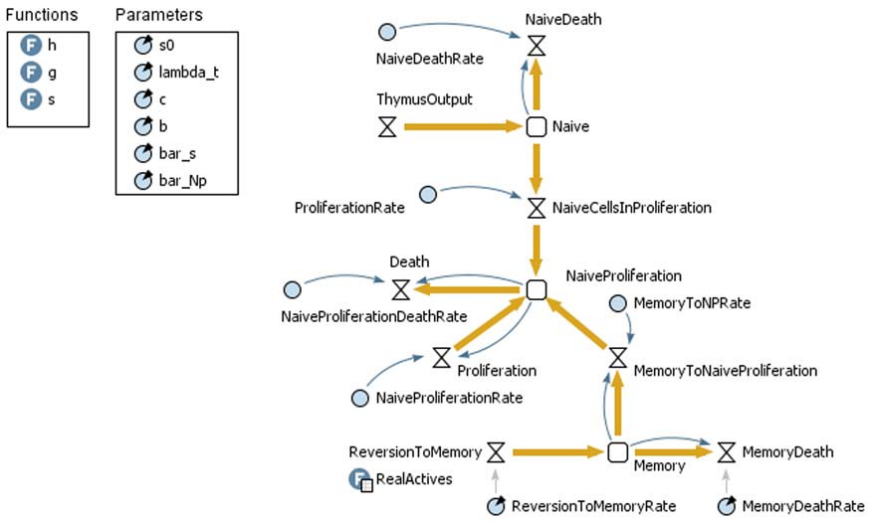
The system dynamics model, functions and parameters.

The SDM’s stock and flow diagram consists of the stocks, flows, information, auxiliaries and parameters, as shown in Fig. 2. In the model, the naïve T cells, naïve T cells from proliferation, and memory cells are the stock variables, as the aim is to store information about how they accumulate overtime.

In the figure, the stock variable that represents the number of naïve T cells (*Naive*) is subject to inflowing thymic output (*ThymusOutput*), and outflows from proliferation (*NaiveCellsInProliferation*) and death (*NaiveDeath*). The inflows for the stock of naïve cells from proliferation’s (*NaiveProliferation*) come from proliferation and reversion from memory (*MemoryToNaiveProliferation*), and the outflow is death, according to Equation 2. The memory stock’s inflow is reversion from active (*ReversionToMemory*). The outflows are reversion to a naïve phenotype (in the figure, *MemoryToNaiveProliferation* and death, as defined by Equation 6). The number of active cells, which is a stock, is given by real-world data of active cells in the human organism (in the figure, it is the table *RealActives*).

Curved arrows in the stock and flow diagram between stocks and flows indicate that there is information about a stock that influences a flow. By looking at Equation 1, it is possible to determine that there is information between the stock *Naive* (*N* in Equation 1) and the flow *NaiveDeath*. For the *NaiveProliferation* stock there is information from it to *Proliferation* and *Death* flows. In the *Memory* stock there is information from it to the flows *MemoryToNaiveProliferation* and *MemoryDeath*. The model parameters are the same as those used the mathematical model.

For our implementation, functions are designed for *s* and *g*, which use the stock *NaiveProliferation* in their calculations. Hence, the information about *NaiveProliferation* is implicit in these functions.

The mathematical parameters and their correspondents in the SD model are shown in Table 2.

**Table 2:** Parameters from the mathematical model and their correspondents from the SD model.

rate	correspondent
*s*_0_	*s*0
*λ*_*t*_	*lambda*_*t*
*c*	*c*
${\bar{N}}_{p}$	*bar*_*Np*
$\bar{s}$	*bar*_*s*
*b*	n
*λ*_*n*_	(Naive) *ProliferationRate*
*μ*_*n*_	*NaiveDeathRate*
*λ*_*mn*_	*MemoryToNPRate*
*μ*_*N*_*p*__	*NPDeathRate*
*μ*_*m*_	*MemoryDeathRate*
*λ*_*a*_	*ReversionToMemoryRate*

Table 3 presents the flows for each stock, their correspondent in the mathematical model and the flow formula. In the table, the functions *s*0(), *s*(), *g*() and *h*() are implemented according to corresponding mathematical functions. The function *time*() returns the current simulation time, which, for this case, is given in years. Furthermore, the *ThymusOuput* is an example of flow which does not have any information or parameter. Hence, it is defined according to the mathematical expression stated.

**Table 3:** Flow calculations for the naïve T cell output model.

Stock	Flow	Expression	Flow formula
*Naive*			*ThymusOutput*	*s*_0_ *e* ^−*λ*_*t*_*t*^ *s*(*N*_*p*_)	*s*0()*e* ^−*λ*_*t*_.*time*()^ *s*()
*NaiveCellsInProliferation*	*λ*_*n*_ *N*	*ProliferationRate*.*Naive*
*NaiveDeath*	*μ*_*n*_ *g*(*N*_*p*_)*N*	(*NaiveDeathRate*.*g*()*Naive*)
*NaiveProliferation*		*Proliferation*	*ch*(*N*, *N*_*p*_)	(*c* × *h*().*NaiveProliferation*)
*Death*	*μ*_*n*_ *N*_*p*_	(*NpDeathRate*.*NaiveProliferation*)
*Memory*			*MemoryToNaiveProliferation*	*λ*_*mn*_ *M*	(*Memory/ToNPRate.Memory*
*ReversionToMemory*	*λ*_*a*_ *A*	(*ReversionToMemoryRate*.*RealActives*(*time*()))
*MemoryDeath*	*μ*_*m*_ *M*	(*MemoryDeathRate*.*Memory*)

### The Agent-based Modelling and Simulation

**Fig 3:**
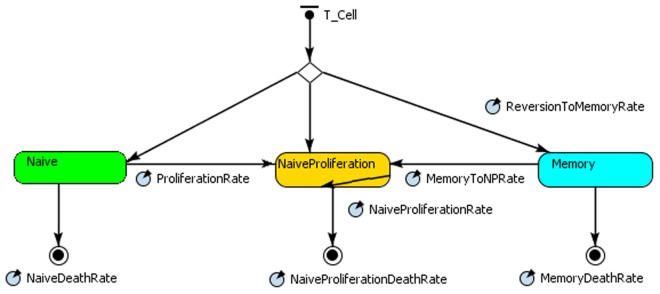
The naïve T cell agent.

In our model T cells are the agents and can assume three states: *Naive*, *NaiveFromProliferation* and *Memory*, as shown in the state chart depicted in Fig. 3. The lozenge at the top of the state chart represents a branch for deciding the T cell current state. In the figure, there are also final states when cells die and are eliminated from the system (represented by a black circle outlined with a smaller filled in circle inside it). Each agent behaviour depends on its current state and occurs according to a certain parameter rate. The agent’s parameters and behaviours corresponding to each state are shown in Table 4.

**Table 4:** Agents’ parameters and behaviours for the naïve T cell output model.

State	Parameters	Reactive behaviour	Proactive behaviour
*Naive*			*NaiveDeathRate*	Dies	
	Is produced by thymus	
*ProliferationRate*		Reproduces
*NaiveProliferation*				*NaiveProliferationDeathRate*	Dies	
*ProliferationRate*	Is produced by Naive proliferation	
*MemoryToNPRate*	Is produced from Memory	
*NaiveProliferationRate*		Reproduces
*Memory*			*MemoryDeathRate*	Dies	
*ReversionToMemoryRate*	Is produced from active cells	
*MemoryToNPRate*		Turns into Naive

The agents’ transitions (arrows) determine the changes in state. The state changes and death rates are given by the ratios defined in the mathematical model. Initially, all the agents are in the *naive* state. As the simulation proceeds, they can assume other stages according to the transition pathways defined in the state chart.

**Fig 4:**
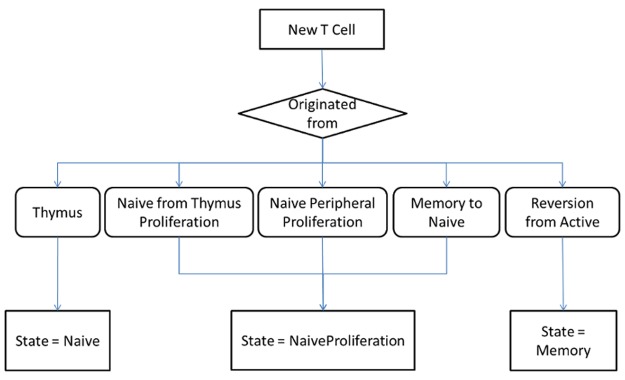
New agent (T cell) state decision flow chart.

When agents reproduce, the newborn agents, which are also T cells, should assume the same state as the parent agent. Apart from proliferation, new agents are also produced from thymic output and reversion from active to memory cells. The algorithm that determines the agent state is given according to the flow chart in Fig. 4.

Our agents respond to changes in time and do not interact with each other directly. For the simulation development, apart from the agents, there is also a function that determines the thymic output and the number of active cells (from the look-up table) that become memory cells. Both are implemented using events that determine when each of these T cells should enter the system. The thymic output calculation, the functions *s*, *g*, *h* and the active cells look-up table are the same as those from the SD model.

### Experiments

Five simulation scenarios were studied, defined by [3] with different values for the parameters. A summary of the parameters used for each scenario is presented in Table 5.

**Table 5:** Simulation parameters for different scenarios. The parameter *c* is only used in the first scenario, where there is no proliferation. In the other scenarios, proliferation is defined by the equation $\left(1+\frac{300}{\bar{N_{p}}}\right)*4.4$ [3].

Scenario	Description	Parameters						
		*λ*_*n*_	*λ*_*mn*_	${\bar{N}}_{p}$	$\bar{s}$	*b*	*μ*_*N*_*p*__	*c*
1	No peripheral proliferation	0.22	0.05	387	0.48	3.4	0.13	0
2	No homeostatic reduction in thymic export, no homeostatic alteration of naive death rate	2.1	0	713	0	0	4.4	–
3	Homeostatic alteration of naive death rate but not thymic export	0.003	0	392	0	4.2	4.4	–
4	Homeostatic alteration of thymic export but no naive death rate	0.005	0	378	2.4	0	4.4	–
5	No restrictions	0.005	0	378	2.2	0.13	4.4	–

The first scenario investigates whether there is the need for naïve peripheral proliferation throughout life to sustain the naïve population. The naïve peripheral proliferation rate for this experiment is therefore set to zero. It also considers reversion from memory to a naïve phenotype.

The second scenario assumes peripheral proliferation with a higher rate of naïve cells becoming naïve proliferating cells (*λ*_*n*_ = 2.1). There is no reversion from memory to a naïve phenotype and no homeostatic reduction in thymic export. The functions *s*, *g* and *h* from the mathematical model are responsible for controlling the thymic export, naïve death rate and naïve peripheral proliferation respectively. In order to change the rate of thymic export, the parameters $\bar{s}$ and ${\bar{N}}_{p}$ are changed. The parameter *b* is set to zero so that the function *g* remains constant during the entire simulation, as does the death rate of naïve cells.

The third scenario alters the function *g* over time by setting the parameter *b* greater than zero (*b* = 4.2). This means that the death rate of naïve T cells from the thymus increases with age as the number of naïve from peripheral proliferations rises. There is no change to the thymic export, no reversion from memory to a naïve phenotype, and the conversion rate of naïve from the thymus to naïve proliferation is low (equal to 0.003).

Scenario 4 is the opposite of scenario 3. In this case there is no change in the death rate of naïve T cells from the thymus. Rather, there is change on the thymic export with time.

Finally, the fifth scenario presents no restrictions, which means that there are changes in thymic export and death of naïve cells over time. Moreover, there is peripheral proliferation and no memory cell turns back to a naïve phenotype.

Two datasets were used for validation of the simulations. Their properties are summarised in Tables 6 (obtained from [3] and [28]) and 7 (obtained from [29]). The data sets contain information about the TREC marker in individuals grouped in age ranges. In the tables, the first column shows the age range of the individuals; the second column has the mean $\frac{\log_{10}TREC}{10^{6}\times nPBMC}$ (peripheral blood mononuclear cell) and the third column contains the number of individuals in each age range.

**Table 6:** The data set used for validation (obtained in [3] and [28]).

Age	$\frac{\log_{10}TREC}{10^{6}\times nPBMC}$	number of individuals
0	5.03	48
1–4	4.93	53
5–9	4.86	19
10–14	4.86	19
15–19	4.56	33
20–24	3.88	26
25–29	3.75	47
30–34	3.61	65
35–39	3.54	73
40–44	3.52	52
45–49	3.37	55
50–54	3.17	16

**Table 7:** The data set collected in Lorenzi *et al*. [29].

Age	$\frac{\log_{10}TREC}{10^{6}\times nPBMC}$	number of individuals
0	4.85	2
1–4	5.29	30
5–9	5.05	33
10–14	4.99	15
15–19	4.56	5
20–24	4.55	12
25–29	4.55	9
30–34	4.44	20
35–39	4.23	15
40–44	4.16	9
45–49	3.82	16
50–54	4.21	21

**Fig 5:**
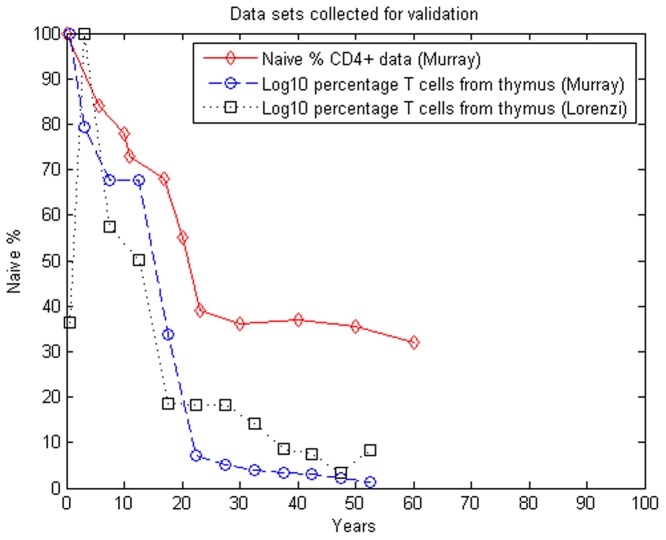
Data sets (collected in [3, 28] and [29]) used for validation of the naïve T cell output simulation models.

Fig. 5 shows the TREC data (naïve from the thymus) and total naïve cell data (provided by [3] [28] and [29]). In the figure, data provided in Table 6 is represented by the symbol ◯; the □ symbol indicates the data from Table 7. In addition, the total percentage of naïve T cells in the body, obtained in [3], is also displayed (symbol ◊).

Each experiment was run for a simulation period of one hundred years taking into account the impact of thymic shrinkage per *mm* ^3^ of peripheral blood, and using 3673 initial naïve cells from the thymus. For the ABM, the simulation was run fifty times and the mean result of these runs was collected.

## Results

**Fig 6:**
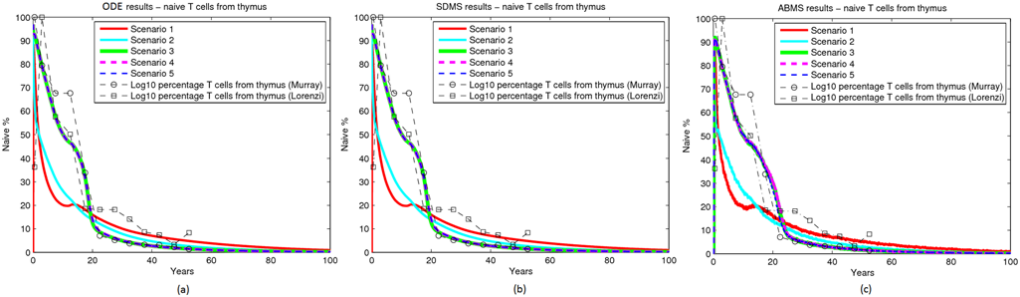
Results for naïve T cells from the thymus.

**Fig 7:**
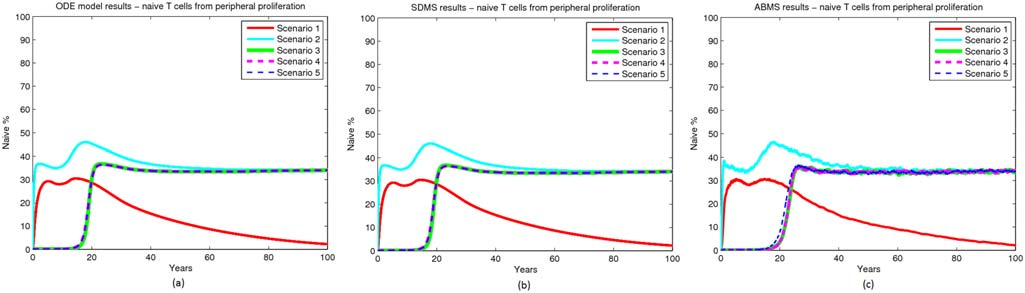
Results for naïve T cells from peripheral proliferation.

**Fig 8:**
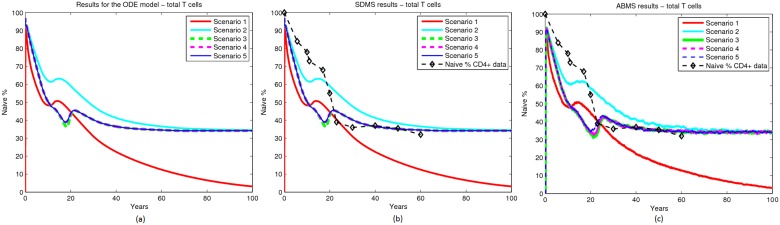
Results for total T cells.

The simulation results contrasting SDMS with ABMS are illustrated in Figs. 6, 7 and 8. Figs. 6(a), 7(a) and 8(a) show the ODE results used as a baseline for our results validation. Overall, when comparing SDMS and ABMS outputs, the results were similar. As expected, the ABMS produced some variation on the simulation curves, while the curve from the SDMS was steady. In addition, SDMS used far less computational resources.

In the first scenario, the results for both simulation techniques show a very similar trend curve, although the ABMS results exhibit a noisier behaviour pattern. Results did not fit the original data Tables (6 and 7). We believe, however that the ABMS results can be improved to match the data more closely. We employed the same laws and rates from the original ODE model, which is one of the shortcomings of our work. We intend in the future to calibrate the ABMS solely with the data provided and observe the changes in the outcome.

Regarding the biological dynamics, naïve cells from the thymus curve demonstrated a substantial decay in thymic export on the beginning of life because of the high death rate. After the twenties, an exponential decay of thymic export was observed and the dynamics followed the thymic decay rate rule defined in the mathematical model. The naïve proliferation curve increased with the decrease of naïve from the thymus, but as there was no proliferation of peripheral cells, they died with no replacement. Thus they followed the same pattern as that of their only source, i.e. thymic naïve cells. The results indicate that peripheral proliferation is important for maintenance of naïve T cells.

Results from scenario 2 matched the original data more closely for both approaches. This case considered peripheral proliferation, as well as a high rate of naïve cells from the thymus turning into peripheral naïve cells. The naïve from the thymus curve shows a substantial decay in the beginning of life because of the death and proliferation rates. On the other hand, the naïve from proliferation curve increased with the decrease of the naïve from the thymus curve. The main difference between these results and the results from the previous scenario is that the number of naïve cells from proliferation reached a stable value after the age of twenty with no further decay. The results indicate the importance of peripheral expansion, but also the need for a smaller rate of naïve to peripheral naïve conversion. Moreover, reversion from memory to a naïve phenotype does not seem to influence the overall quantities of cells.

Scenario 3 took into account the results produced in the previous scenarios and adjusted the parameters so that a more accurate output was obtained. The naïve from the thymus curve showed a decay at the beginning of life followed by an interval of stability. By the age of twenty the thymic export decreased in an exponential trend. With the decay of naïve from the thymus, the naïve repertoire changed from the thymic source to the peripheral proliferation source. By performing these simulations it is therefore possible to gain understanding of how the decay of naïve cells occurs over time. In this scenario the results closely matched the original data. Scenarios 4 and 5 produced similar results to scenario 3. This indicates that alterations in thymic export and in naïve death do not interfere significantly with the overall dynamics of the naïve T cells.

In the five scenarios studied, the simulations produced similar results for both SDMS and ABMS. This can also be observed in the results of Wilcoxon rank sum tests applied to both the ABMS and the SDMS results (Table 8). The table reports p-values associated with Wilcoxon rank sum tests for the five scenarios. Our hypothesis is that the outcomes produced are not significantly different. The p-values for each test all exceed the 0.05 (5%) significance level, indicating that the distributions of the outcomes of the various simulation approaches are not statistically different and therefore, the tests failed to reject the similarity hypothesis.

**Table 8:** Wilcoxon test with 5% significance level comparing the results from SDMS and ABMS.

Scenario	p
1	0.8650
2	0.8750
3	0.7987
4	0.8408
5	0.9719

## Discussion

Besides mathematics, simulation becomes more and more popular in immunology research. However, comparisons of different simulation methods in this field are very rare. In this research we contrasted two different simulation methods, SDMS and ABMS, for an immunosenescence case study, and tested whether they provided a different insight. Our simulation models were based on mathematical equations (describing thymic decay and naïve cells dynamics) converted into SDMS and ABMS. Five simulation scenarios were studied investigating different sustaining sources for the naïve cell population. Our research questions were: Which simulation approach is more suitable for this problem? Can these approaches be employed interchangeably? Is there any benefit in using one approach rather than the other?

Results for both methods matched those from the original ODE model. In the ABMS simulation, cells were subject to individual rates that occurred during the time slot in which they were created. However, this did not seem to have influenced the final outcome. Further, as our experiments involved large populations, the variability inherent in ABMS did not considerably affect the overall dynamics of the simulated population. In the SDMS simulation outputs were exactly the same as those from the ODEs, as expected. The SDMS was simpler to implement and required significantly less computational resources such as memory, processing time and complexity. Therefore, although in this work both approaches can be employed interchangeably, SDMS seems more suitable.

The SDM and ABM were built based on the initial mathematical rules used to fit the data collected, which is a limitation of our work. This induces the simulations to behave similarly to the ODE model simulations, meaning that the new results were no more informative than the original ODE model, as they fit the data in the same manner. Furthermore, incorporating the functions for the thymus output, *g*, *s* and *h* into the ABM made it hybrid; and its stochastic character did not produce any significant variant (extreme pattern) of the expected outcomes. Emergence was also not observed.

Another important point is that the agents considered are static (no movement) and non-interacting, which was a limitation imposed by the way the biological system was described, i.e. the features of the problem studied did not allow us to exploit the ABMS capabilities appropriately.

The advantage of applying SDM and ABM to this case study is mostly that the modelling processes are more intuitive than the original ODE model. Additionally, the diagrams of both SDM and ABM are easier to communicate in multidisciplinary contexts. In particular, due to the characteristics of the problem, SDM also appears to be more suitable for the modelling process. We believe therefore that the value of employing simulation in this case study context is in model communication to non-experts. Our future goal is to find ways to formalise the translation from ODE to SDM and ABM and to find out which of these is actually preferred by practitioners.

As another future aim, to overcome our modelling and simulation shortcomings, it is our intention to rebuild and calibrate our models based solely on the data provided. Subsequently, we intend to assess and report on the impacts of this procedure. We hypothesize that changes in the SDMS and ABMS can show further differences in the results. In addition, we believe we can achieve a better representation of the biological phenomena and therefore better fitting of the data. We intend to subsequently validate our modelling efforts with imunologists. This should help in enforcing the message that, as well as traditional mathematical modelling, SDM and ABM simulations are also very useful for understanding immunosenescence.
